# Effect of Microimplant Neck Design with and without Microthread on Pullout Strength and Destruction Volume

**DOI:** 10.3390/ma14205991

**Published:** 2021-10-12

**Authors:** Yu-Chuan Tseng, Han-Sheng Chen, Szu-Yu Hsiao, Kun-Jung Hsu, Chun-Ming Chen

**Affiliations:** 1 School of Dentistry, College of Dental Medicine, Kaohsiung Medical University, Kaohsiung 807, Taiwan; yct79d@gmail.com; 2 Division of Orthodontics, Department of Dentistry, Kaohsiung Medical University Hospital, Kaohsiung 807, Taiwan; 3 Department of Dentistry, Kaohsiung Municipal Siaogang Hospital, Kaohsiung 812, Taiwan; tabbyguy@yahoo.com; 4 Division of Dentistry for Child and Special Needs, Kaohsiung Medical University Hospital, Kaohsiung 807, Taiwan; 5 Department of Dentistry, Kaohsiung Medical University Hospital, Kaohsiung 807, Taiwan; 6 Division of Oral and Maxillofacial Surgery, Department of Dentistry, Kaohsiung Medical University Hospital, Kaohsiung 807, Taiwan

**Keywords:** microthread neck, pullout strength, destruction volume, microimplant

## Abstract

The microthread neck concept has been applied to dental implants. This study investigated the pullout strength and destruction volume of orthodontic microimplants with and without the microthread neck design. Fifteen microimplants (diameter: 1.5 × 10 mm) of three types (Types A and B: without microimplant neck; Type C: with microimplant neck) were tested. The insertion torque (IT), Periotest value (PTV), horizontal pullout strength (HPS), and horizontal destruction volume (HDV) of each type were measured. Kruskal–Wallis H test and Dunn’s post-hoc comparison test were performed to compare the measured values of the three types of microimplants. The correlations of the measured values were used to perform the Spearman’s correlation coefficient analysis. The ITs of Types B (8.8 Ncm) and C (8.9 Ncm) were significantly higher than those of Type A (5.2 Ncm). Type B yielded the lowest PTV (4.1), and no statistical differences in PTV were observed among the three types. Type A had a significantly lower HPS (158.8 Ncm) than Types B (226.9 Ncm) and C (212.8 Ncm). The three types did not exhibit any significant differences in the HDV. The results of the Spearman’s correlation coefficient test revealed that HDV (ρ = 0.710) and IT (ρ = 0.813) were strongly correlated with HPS, whereas for PTV and HPS, it was not. HPS was strongly and significantly correlated with HDV. The orthodontic microimplant with a microimplant neck design did not perform better than that without a microthread in the mechanical strength test.

## 1. Introduction

The control of unwanted tooth movement during orthodontic treatment is challenging, especially in cases of complex malocclusion. Microimplants in the maxilla and mandible are a form of skeletal anchorage that aid in complex orthodontic treatment [1,2]. Because of their ability to control tooth movement selectively and efficiently, microimplants have become increasingly prevalent in orthodontic treatment. The stability of microimplants can be divided into two types: primary and secondary. Primary stability refers to mechanical interlocking between a microimplant and the surrounding bone, which is a short-term phenomenon. Secondary stability refers to a biological phenomenon in which the bone surrounding the microimplant is modified shortly after implant placement and the new bone formed attaches to the microimplant. Secondary stability is the outcome of long-term bone healing and osseointegration. Orthodontic force is usually applied to microimplants immediately or within 1 month of placement to move the teeth. Therefore, primary stability is more crucial than secondary stability.

The mechanical properties of a microimplant are key factors that determine its stability and success. Several factors, such as insertion torque (IT) [3], removal torque [4], microimplant geometry [5], and pullout strength [6,7,8], can be measured to determine the stability of microimplants. The reason for microimplant failure in the initial stage is that the mechanical interlocking strength between the bone and the microimplant weakens after orthodontic force is applied. Therefore, less invasive methods [9,10] have been proposed to assess the stability of microimplants. One of these methods [11,12] involves the Periotest M (Medzintechnik Gulden, Modautal, Germany), which has been used successfully. This study used the Periotest value (PTV) to determine the primary stability of the microimplants.

In an experimental study, Rasmusson et al. [13] investigated the integration and stability of grit-blasted implants with retention elements (microthreads) on the implant neck. They found that microthread implants showed a higher degree of bone-implant contact and a higher level of bone regenerated at defect sites, as compared with the other implants. Resonance frequency analysis demonstrated a significantly higher increase in microthread implants compared with their control groups. The microthread design of an implant neck has been reported to reduce marginal bone loss and increase the long-term survival of dental implants [14]. During mastication, most of the masticatory force on the dental implant is applied vertically. Unlike the force on dental implants, orthodontic force is mostly horizontal rather than vertical, which raises the question of whether the microthread design in implant necks significantly improves the stability of orthodontic micro implants. The destruction volume is a measure of the damage caused to the artificial bone after a pullout test. However, no study has explored the correlation between destruction volume and pullout strength. This study evaluated the primary stability, mechanical properties, and destruction volume of microimplants (with and without microthread neck design). The null hypothesis was that horizontal pullout strength (HPS) and horizontal destruction volume (HDV) are not significantly correlated.

## 2. Materials and Methods

**Figure 1:**
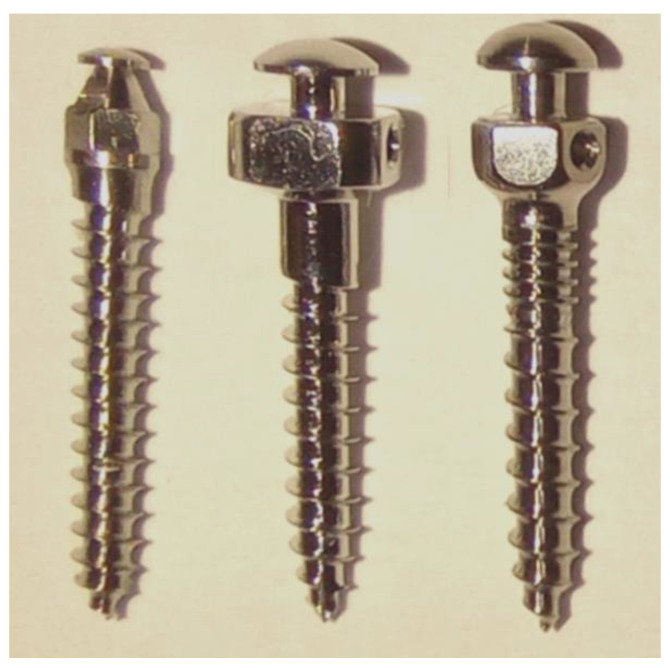
Microimplants manufactured with 3 designed types, from left to right: Type A. 5 × 10 mm; no microthread neck), Type B (1.5 × 10 mm; no microthread neck), and Type C (1.5 × 10 mm; microthread neck).

Three types of microimplant (Types A, B, and C) were analyzed in this study (Figure 1). Type A (1.5 × 10 mm, titanium alloy) and B (1.5 × 10 mm, stainless steel) did not have a microthread on the neck. Type C (1.5 × 10 mm, titanium alloy) had a 2-mm microthread on the neck. This study used 15 microimplants—five of each of the three types of microimplants—and tested their horizontal pullout strengths. To account for the thickness of the gingiva and cortical bone, the microimplants were inserted into the artificial bone (Sawbones, Pacific Research Laboratories, Inc., Vashon Island, WA, USA) at a depth of 8 mm. The artificial bone is composed of a 2-mm cortical bone (40 pcf) and bone marrow (20 pcf).

**Figure 2:**
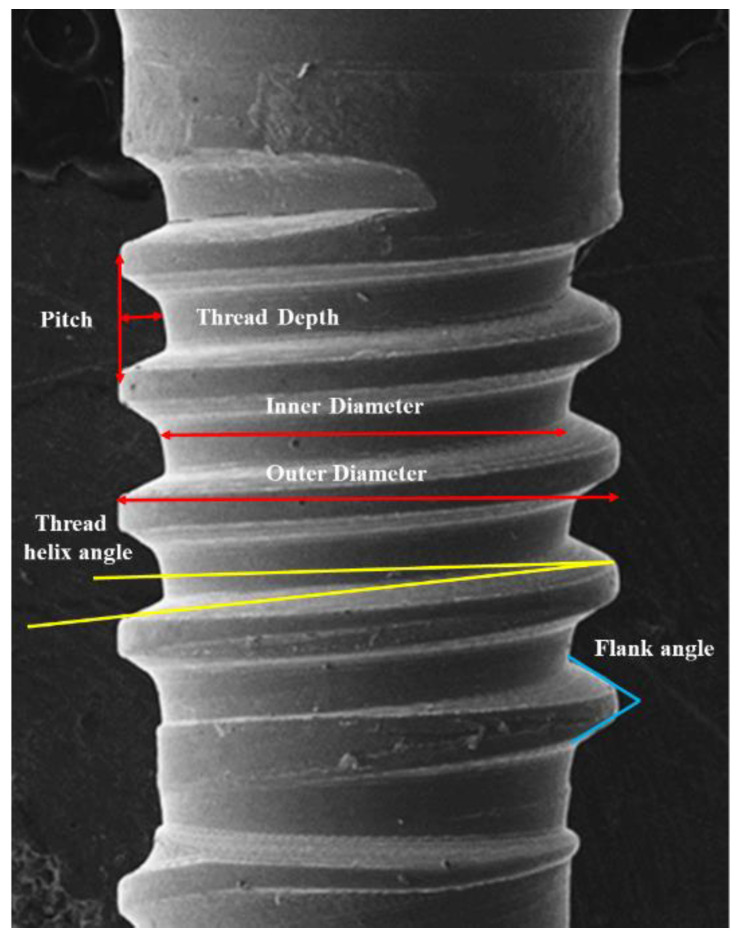
Surface features of thread (Type C; microthread neck) using scanning electron microscopy (15 kV × 30, Hitachi SU8010, Japan). Red double-headed arrows: dimensions of thread. Yellow: flank angle. Blue: thread helix angle.

**Figure 3:**
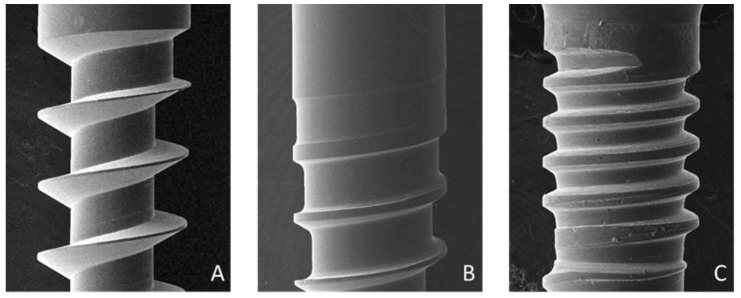
Thread in the neck portions of microimplants were magnified by a scanning electron microscope (Hitachi SU8010, Japan). From left to right as follow: Type (**A**) (no microthread neck), Type (**B**) (no microthread neck), and Type (**C**) (microthread neck).

**Figure 4:**
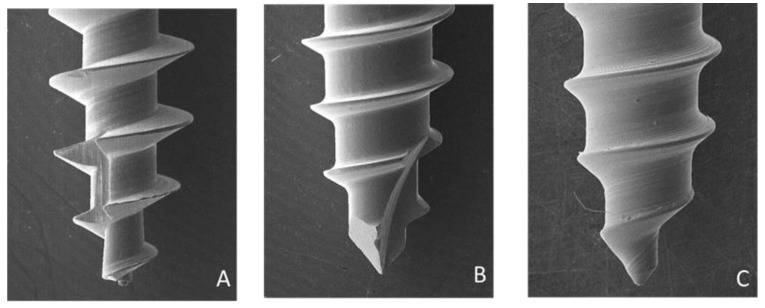
Thread in the apical portions of microimplants were magnified by a scanning electron microscope (Hitachi SU8010, Japan). From left to right: Type (**A**) (no microthread neck), Type (**B**) (no microthread neck), and Type (**C**) (microthread neck).

**Figure 5:**
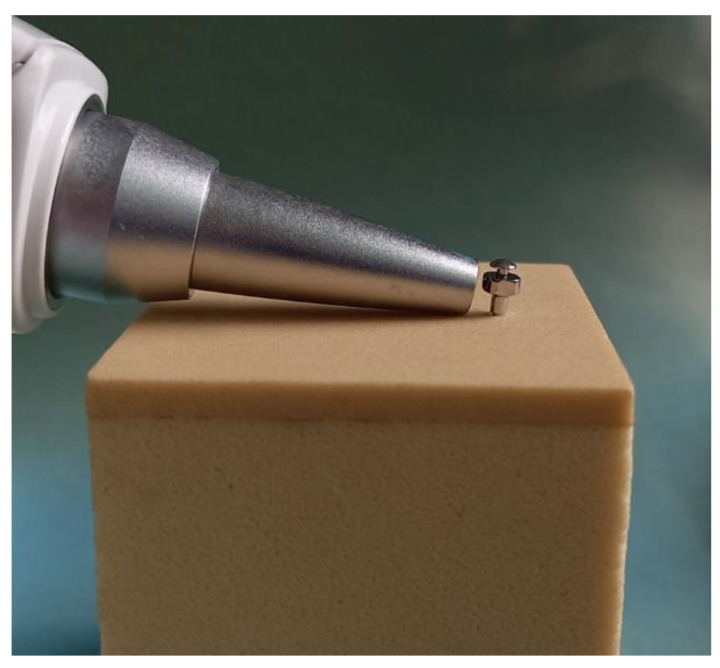
Tapping head device of the Periotest system (Periotest M, Medzintechnik Gulden, Modautal, Germany) to percuss the microimplant.

**Figure 6:**
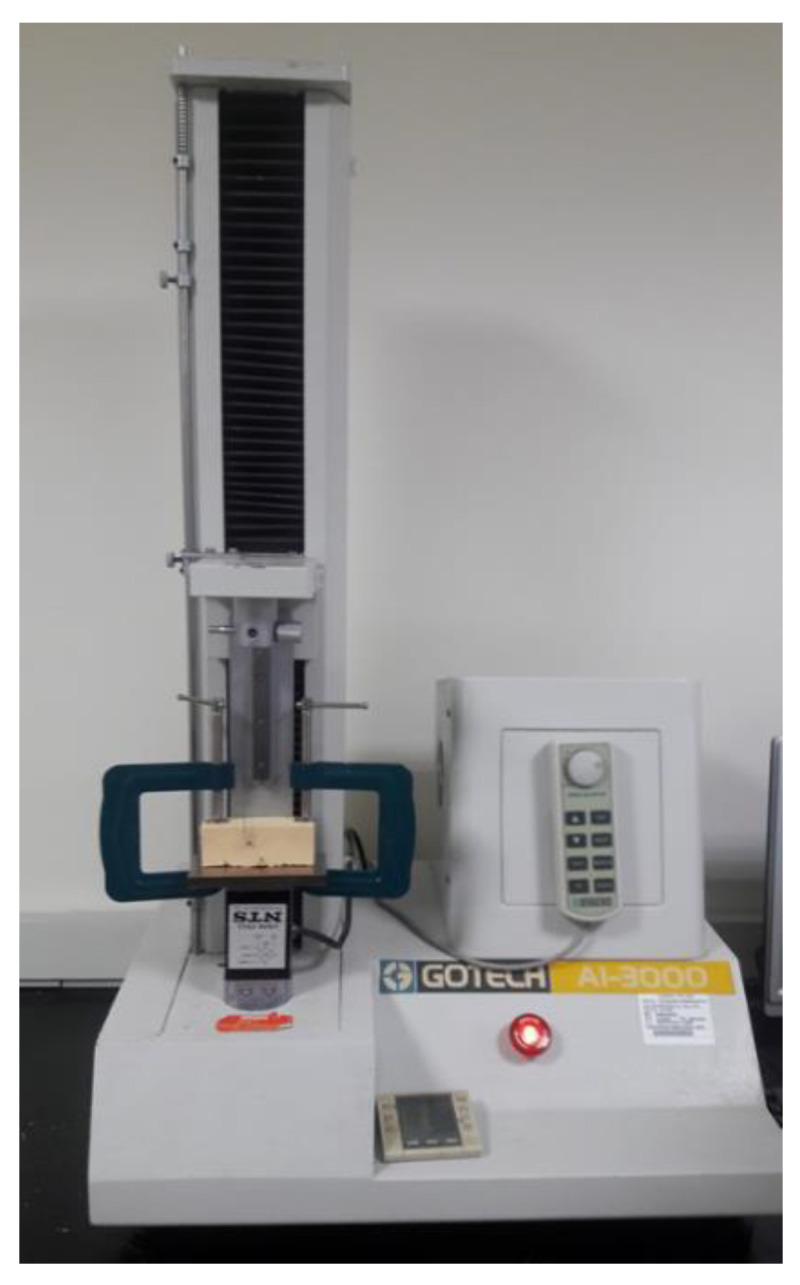
An orthodontic wire (0.018 inch) was passed through the hole of microimplant, then tied into the material testing machine (GOTECH AI-3000, Gotech Testing Machines Inc., Taichung, Taiwan).

The surface features of the microimplant thread (Figure 2, Figure 3 and Figure 4) were examined using a scanning electron microscope (Hitachi SU8010, Tokyo, Japan). The weights of the microimplants were measured using an analytical balance (AS 220/C1, Radwag, Poland). A torque meter (Lutron Electronic Enterprise Co., Ltd., Taipei, Taiwan) was used to record the insertion torque of the microimplants. The microimplants were percussed using a wireless Periotest M, which is an electric tapping machine, and their PTVs were recorded (Figure 5). HPS was determined using a material tester (GOTECH AI-3000, Gotech Testing Machines Inc., Taichung, Taiwan) via a 0.018-inch orthodontic arch-wire tied to a hole at the neck of the microimplant (Figure 6). After the HPS of the microimplant was measured, the remnant hole (horizontal destruction volume) of the artificial bone was filled with a 3M Filtek Supreme Flowable resin (St. Paul, MN, USA; density: 1.5 g/cm^3^) and weighed using an analytical balance. The HDV was calculated using mass–density conversion.

Statistical analysis was performed using SPSS software (IBM Corporation, Armonk, NY, USA), and a *p* value of less than 0.05 was considered statistically significant. Kruskal–Wallis H test and Dunn’s post-hoc comparison test were performed to compare the measured values of the three types of microimplants. The correlations of the measured values were used to perform Spearman’s rho correlation coefficient and nonparametric analysis. The strengths of the correlations were as follows: very weak (0–0.19), weak (0.20–0.39), moderate (0.40–0.59), strong (0.60–0.79), and very strong (0.80–1).

## 3. Results

Table 1 presents the dimensions and characteristics of the microimplants. Type B (0.79 mm) had the smallest inner diameter, and Type C had the largest neck (1.24 mm) and middle (1.20 mm) region. The inner–outer diameter ratios of the microimplants were 0.53 for Type A, 0.67 for Type B, 0.79 at the neck of Type C, and 0.80 in the middle of Type C. Type A had the largest thread depth (0.35 mm) and Type C had the smallest thread depth (0.16 and 0.15 mm at the neck and in the middle, respectively). Type C had the greatest flank angle at the neck (58.95°) and middle (66.87°). Type A had the greatest thread helix angle (15.62°).

Type B (211.7 mg) was considerably heavier than Type A (57.1 mg) (Table 2). Type A had the smallest IT (5.2 Ncm) among the three types, which indicates that its placement required the least amount of effort. Types B and C yielded similar IT values (8.8 Ncm and 8.9 Ncm, respectively). The ITs of Types B and C were considerably higher than those of Type A. Type B yielded the lowest PTV (4.1), and no statistical difference in PTV was observed among the three types. Type A had the lowest HPS (158.8 Ncm) among the three types, and significant differences were observed among the three types. The HPSs of Type B (226.9 Ncm) and Type C (212.8 Ncm) were significantly higher than those of Type A (158.8 Ncm). No significant differences in HDV were observed among the three types.

As Table 3 shows, the primary stability decreases as the PTV/IT increases. Type A had the highest PTV/IT value (1.0), which was significantly higher than that of Type B (0.5) and Type C (0.6). Type B, which had the lowest PTV/IT value, had the highest primary stability. No significant differences in HDV/IT, HPS/IT, and HDV/PTV were observed among the three types. The HPS/PTV of Type B (58.9) was significantly higher than that of Type A (30.9). The HPS/HDV value of Type B (8.3) was significantly higher than that of Type A (6.8). The results of the Spearman’s rho correlation coefficient test (Table 4) revealed that IT had a negative moderate correlation (ρ = −0.516) with PTV. This implies that a higher IT value has a lower PTV value (better primary stability). IT had a strong positive (ρ = 0.637) value and very strong (ρ = 0.813) correlations with HDV and HPS (greater secondary stability). PTV had a moderate negative correlation (ρ = −0.569) with HDV. This suggests that a higher PTV (less primary stability) has a lower HDV value. The PTV had no significant correlation with HPS, indicating that the primary stability cannot infer secondary stability. HPS had a strong positive correlation (ρ = 0.710) with the HDV, implying that a higher secondary stability (HPS) has a larger HDV. Therefore, the null hypothesis was rejected.

## 4. Discussion

To provide strong orthodontic anchorage, microimplants must withstand orthodontic force and remain in a stable position in the bone until they are no longer required. The stability of microimplants depends on biological changes in the surrounding bone and is crucial to the overall performance. Primary stability is a mechanical phenomenon derived from the connection between the microimplant and the bone. The design of microimplants affects primary stability. Threads are designed to facilitate placement, prevent loosening, provide strength, and withstand multiple dimensional loads. Studies [5,15] have demonstrated that characteristics such as length, diameter, thread depth, thread design, and thread pitch are crucial in determining the holding power. IT is the most commonly used property to measure the mechanical strength of microimplants. IT is the first mechanical property to occur during the placement of a microimplant.

To achieve a higher success rate, Motoyoshi et al. [16] recommended the use of a 1.6-mm microimplant with an IT ranging from 5 to 10 Ncm. IT exceeding this range can cause peeling-off of the surrounding bone and a reduction in microimplant retention, which can result in microimplant failure. The three types of microimplants used in our study had mean IT values of less than 10 Ncm. Geometric shape is a significant factor in the biomechanics of microimplants, and changes in these factors can affect the mechanical strength. The flutes of microimplants are the longitudinal channels on the threaded part of the screw, which is used to cut and remove bone fragments. Flutes vary in length and number and are a common feature in self-tapping microimplants. Type A had the longest flute length and the lowest ratio of inner to outer diameter among the three types, which contributed to its lowest IT value. This indicates that the placement of Type A requires the least amount of effort. As for differences between microimplants with and without microthread neck designs, no difference in IT was observed between Types B and C.

Dental implants with lower thread depth in the neck are commonly used for cortical bone, whereas those with higher thread depth are better suited for spongy bone. A higher thread depth can provide more holding power because it increases the volume of bone between the threads, as well as the contact between the bone and the implant. Unlike invasive methods, noninvasive methods measure implant stability without disrupting the bone–implant interface. Therefore, noninvasive methods can be used to measure changes in the stability of microimplants over time. The Periotest method evaluates dental implant stability using an electromagnetic tapping rod and an accelerometer, and is a reliable method for determining the stability of implants. The Periotest method can be used to evaluate the stability of dental implants and orthodontic microimplants, with PTVs ranging from −8 to 50. We determined that the PTV is related to the weight and thread design of the microimplant. The weights of the microimplants vary depending on the material. Type B, made of stainless steel, is the heaviest of all; Types A and C are made of titanium alloy. Stainless steel weighs considerably more than titanium alloys; therefore, stainless steel yields a lower PTV when tapped by a Periotest device than titanium alloy. The thread helix angles of the microimplants were ranked in the following order: Type B < Type C < Type A. Therefore, Type B had the smallest PTV among the three types of microimplants. Bone density is also a key factor in the primary stability of microimplants. Type C is outfitted with a microthreaded neck design to engage the cortical bone and improve primary stability. However, Type C did not decrease the PTV, or exhibit superior primary stability, in comparison to Type B (without the microthread neck design).

Pullout strength is commonly used to evaluate the primary stability of orthodontic microimplants. Studies have confirmed that bone density is positively correlated with pullout strength [17,18], and suggested that high bone density indicates superior primary stability. Chapman et al. [19] determined that increasing the thread depth using a porous material produced a greater pullout strength and pullout resistance. The thickness of the cortical bone has been positively correlated with the primary stability of an implant [7,8]. In the HPS test, we discovered that the cortical bone must be destroyed before the microimplant can be pulled out. The force required to pull the microimplant from the bone increases with the cortical thickness. Therefore, the inner–outer diameter ratio was highly influential. Type A had a relatively small inner diameter and exhibited a relatively large curvature when pulled out. Its small inner diameter facilitated the pullout process and yielded the lowest HPS value among all the three types. Types B and C exhibited relatively small curvatures during the pullout process, whereas Type B was scarcely bent, which was likely due to the relatively high hardness of stainless steel. In addition, the microthread portion of Type C had a larger inner diameter and a smaller curvature than the other types. Therefore, the HPS of Types B and C was significantly higher than that of Type A. However, although Type C had a microthreaded neck design, no difference in HPS was observed in Type B. Greater trauma leads to a greater amount of damage. Therefore, damage to the artificial bone (HDV) increased with the HPS value. However, no similar phenomenon was observed for HDV. The HPS of Type A was significantly lower, but its HDV value was not different from that of Types B and C. In addition, no difference between Type C (microthread neck design) and Types A and B was observed in terms of the HDV value.

Compared with Type A, Types B and C had significantly lower PTV/IT values, which indicates that Type A requires the least IT but has the lowest primary stability. During the immediate application of microimplant orthodontic force, maintaining a high primary stability is more crucial than reducing IT. No significant differences were observed among the three types in terms of HPS/IT and HDV/IT. This indicates that IT is unsuitable for inferring the dynamic retention force of microimplants. Because the three types had similar PTVs and HDVs, no significant difference was observed in terms of HDV/PTV. This result indicates that the PTV is not an appropriate measurement for interpreting HDV. However, the HPS/PTV of Type B (58.9) was significantly larger than that of Type A (30.9). Therefore, the PTV could reflect dynamic retention forces, such as the HPS. The HPS/HDV of Type B (8.3) was significantly larger than that of Type A (6.8). Therefore, a larger degree of destruction was caused by a greater force.

The thread design (consisting of length, diameter, and shape) strongly influences primary and secondary stability. With regard to the significant correlation between IT and other test values (PTV, HPS, and HDV), IT had a negative moderate correlation (ρ = −0.516) with PTV, a strong positive correlation (ρ = 0.637) with HDV, and a very strong (ρ = 0.813) correlation with HPS. This implies that IT can infer PTV, HPS, and HDV. Higher IT values signify lower PTV values (better primary stability) and larger HPS values (greater primary stability). However, the IT must be less than 10 cm to prevent damage to the surrounding bone. PTV exhibited no significant correlation with HPS. We discovered that the static retention force (PTV) is different from the dynamic retention force (HPS). Because destruction is a continuous process, PT is not an appropriate predictor of HPS. PTV had a moderate negative correlation (ρ = −0.569) with HDV, implying that a higher PTV (less primary stability) has a lower HDV value. HDV is a remnant of destruction resulting from the HPS process. In this study, HDV had a strongly significant correlation (0.710) with HPS. Therefore, HDV is an appropriate tool for determining the mechanical strength (IT, PTV, HPS) of microimplants.

The fatigue of the material is gradually deformed owing to cyclic loading. The cyclic fatigue of an implant, from initiation to propagation of cracks, can be defined as the stress, strain, and deformation induced in an implant. Song et al. [20] found that the implant diameter had an effect on the ability to withstand both static and cyclic loads. The ultimate failure load and fatigue cycles decreased as the implant diameter decreased. Kim et al. [21] compared the fatigue characteristics and fracture patterns between single- and multi-directional loading on a dental implant. They found that the multi-directional loading mode in the worst-case environment can reproduce the vertical fracture pattern. The interlocking strength and cyclic fatigue strength are two important mechanical properties of a microimplant. In this study, we investigated the interlocking strength (IT, PTV, HPS, and HDV) between the bone and the microimplant. Cyclic fatigue weakens the microimplant and needs to be studied further. Present study had some limitations in the experimental design. First, sample size was only 15 microimplants (5 of each type) and it was not evident enough to provide clinical consideration. Another limitation was that three types of the microimplants had not only different neck designs but also different geometric shapes including different thread depth, pitch, and angle, etc. Further research should consider the designs of microimplants in conformity and differences.

## 5. Conclusions

Orthodontic microimplants differ in several aspects, including geometric shape, material, size, and implant placement technique (e.g., the presence or absence of pilot holes). The high primary stability of microimplants is critical in orthodontics because it enables orthodontists to perform the loading of microimplants immediately after placement, which improves efficiency. Secondary stability is related to the remodeling and formation of new bones around the microimplant over time. Their correlation must be considered, and insufficient primary stability and bone support can reduce secondary stability. In conclusion, the pullout strength test is a dynamic and destructive process. Pullout tests result in a destruction volume. In this study, HPS was strongly and significantly correlated with the HDV. Orthodontic microimplants with microthread neck designs did not outperform those without microthreads in the mechanical strength test.

## Figures and Tables

**Table 1:** The parameters of microimplants.

Type	A	B	C	
	Middle	Middle	Neck	Middle
Outer diameter (mm)	1.50	1.57	1.56	1.50
Inner diameter (mm)	0.79	1.05	1.24	1.20
Inner/Outer diameter ratio	0.53	0.67	0.79	0.80
Thread pitch (mm)	0.76	0.73	0.40	0.77
Thread depth (mm)	0.35	0.26	0.16	0.15
Flank angle; degree	43.19	42.04	58.95	66.87
Thread helix angle; degree	15.62	12.17	7.07	12.99

Type A, Type B: without microthread neck; Type C: with microthread neck.

**Table 2:** Mechanical strengths (IT, PTV, HPS, HDV) analysis of microimplants in the Kruskal-Wallis H-test with a Dunn’s post hoc comparison test.

Micro-Implants	Weight		IT		PTV		HPS		HDV	
	Mean	SD	Mean	SD	Mean	SD	Mean	SD	Mean	SD
A	57.1	0.20	5.2	0.69	5.3	0.98	158.8	9.42	26.4	1.41
B	211.7	2.10	8.8	2.32	4.1	1.03	226.9	12.64	27.3	1.40
C	108.1	0.25	8.9	2.19	4.9	0.85	212.8	24.55	23.6	2.69
Intergroup	B > A		B > A, C > A		―		B > A, C > A		―	
Comparison *										

* Statistical significance, *p* < 0.05; ―: not significant. Weight (mg); IT: (insertion torque, Ncm); PTV: (Periotest value). HPS: (horizontal pullout strength, Ncm); HDV: (horizontal destruction volume, mm^3^).

**Table 3:** Mechanical strength ratios (PTV/IT, HDV/IT, HPS/IT, HDV/PTV, HPS/PTV, HPS/HDV) analysis of microimplants in the Kruskal-Wallis H-test with a Dunn’s post hoc comparison test.

Micro-Implants	PTV/IT		HDV/IT		HPS/IT		HDV/PTV		HPS/PTV		HPS/HDV	
	Mean	SD	Mean	SD	Mean	SD	Mean	SD	Mean	SD	Mean	SD
A	1.0	0.27	4.6	0.98	30.7	3.32	4.6	0.90	30.9	5.58	6.8	0.72
B	0.5	0.17	3.2	0.68	27.0	5.80	7.0	1.86	58.9	17.13	8.3	0.29
C	0.6	0.18	3.1	0.55	24.8	4.30	5.6	1.13	45.0	9.67	8.0	0.74
Intergroup	A > B, A > C		―		―		―		B > A		B > A	
comparison *												

* Statistical significance, *p* < 0.05; ―: not significant. Weight (mg); IT: (insertion torque, Ncm); PTV: (Periotest value). HPS: (horizontal pullout strength, Ncm); HDV: (horizontal destruction volume, mm^3^).

**Table 4:** Spearman’s rho correlation coefficient for hypothesis test.

Variables	IT	PTV	HDV	HPS
IT	1	−0.516 *	0.637 *	0.813 *
PTV	−0.516 *	1	−0.569 *	−0.460
HDV	0.637 *	−0.569 *	1	0.710 *
HPS	0.813 *	−0.46	0.710 *	1

*: Statistical significance: *p* < 0.05. IT: Insertion torque, Ncm; PTV: Periotest value. HPS: Horizontal pullout strength, Ncm. HDV: Horizontal destruction volume, mm^3^.

## Data Availability

The data used to support the findings of this study are included in this article. The data used to support the findings of this study are available from the corresponding author upon request.
